# Does Green-Person-Organization Fit Predict Intrinsic Need Satisfaction and Workplace Engagement?

**DOI:** 10.3389/fpsyg.2019.02285

**Published:** 2019-10-16

**Authors:** Carol Hicklenton, Donald William Hine, Natasha Maria Loi

**Affiliations:** Faculty of Medicine and Health, School of Psychology, University of New England, Armidale, NSW, Australia

**Keywords:** pro-environmental work climate, environmental values, person-organization fit, intrinsic need satisfaction, work withdrawal, workplace engagement

## Abstract

The current study assessed whether high green-person-organization fit (GPO; the extent to which an organization’s commitment to pro-environmental outcomes is congruent with its employees’ environmental values) predicts employees’ intrinsic need satisfaction and engagement in the workplace. The sample consisted of 818 full-time Australian workers, which is sourced from an online panel. Consistent with the GPO model, pro-environmental work climate was a more potent predictor of intrinsic need satisfaction and engagement for employees with strong ecocentric values than those with weak ecocentric values. Mediation analyses revealed that the effect of work climate on employee engagement was fully mediated by intrinsic need satisfaction, and this effect was strongest when GPO fit was high. Overall, our findings suggest that organizations with pro-environmental work climates that match their employees’ values have more satisfied and committed workforces.

## Introduction

Strategies to motivate and retain valued employees are crucial for organizational success (Gagné and Panaccio, 2015). More than ever, organizations expect their employees “to be proactive and show initiative, collaborate smoothly with others, take responsibility for their own professional development, and be committed to high quality performance standards” (Bakker and Schaufeli, 2008, p. 147). For this to happen, organizations need engaged workers. Many organizations offer a competitive salary and benefits as incentives. However, research on workplace engagement has shown that extrinsic benefits such as pay and promotion may be less important to workers than positive work climates characterized by polices, practices, and procedures that align with employees’ personal values and beliefs (Deci and Ryan, 2015). This message is resonating with corporate decision makers. A report by PwC (2014), based on a survey of 500 HR professionals, indicated that 36% of responding organizations were developing strategies to enact climates of corporate responsibility that match employees’ values and beliefs. Using a large sample of employed Australians, the current study combines person-environment fit with self-determination theory to determine whether the match between organizations’ pro-environmental work climates and employees’ pro-environmental values predicts employee engagement in the workplace.

### Work Climate

Work climate can be defined as employees’ perceptions of their organization in terms of its policies, practices, and procedures (Schneider et al., 2013). Work climate is similar to work culture in the sense that both terms are used to describe the “character” of working environments. But, they emerge from different academic traditions; culture from anthropology, and climate from Lewinian psychology (Schneider, 1990). An organization’s culture reflects the underlying assumptions that shape its operations, encompassing embedded narratives and symbols that are largely taken for granted and guiding behavior primarily at a subconscious level. Climate, on the other hand, reflects more surface-level processes and practices to which employees consciously attend (Kuenzi and Schminke, 2009).

Work climate has important effects on organizations and the people they employ (Kuenzi and Schminke, 2009). It drives employee attitudes and behavior by directing employee performance and enforcing normative standards (Schneider, 2000; Zohar and Luria, 2005). Previous research has linked positive work climates – the presence or absence of polices, practices, and procedures that support corporate responsibility – to a range of workplace behaviors including organizational citizenship (Ehrhart, 2004), safety (Clarke, 2006), ethics (Martin and Cullen, 2006), and performance of both individuals (McKay et al., 2008) and teams (Colquitt et al., 2002).

In an influential review of work climate research, Kuenzi and Schminke (2009) found that positive work climates also elicit higher levels of employee engagement. For example, perceptions of a strong climate for justice were associated with lower turnover intentions (Simons and Roberson, 2003), and perceptions of ethical climates have been linked to employee job satisfaction, commitment, and retention (Cullen et al., 2003; Ambrose et al., 2008). Similar effects have been observed for pro-environmental work climates, with several studies finding that organizations with such climates have more satisfied (Tilleman, 2012) and committed workers (Spanjol et al., 2015) who are less likely to search for new jobs elsewhere (Lamm et al., 2015).

In summary, work climates with policies, practices, and procedures that reflect a commitment to corporate responsibility appear to increase employee satisfaction and engagement. Nevertheless, some studies suggest that not all employees respond to work climates in the same way (Kuenzi and Schminke, 2009). Climates that advocate ethics, justice, and/or pro-environmental outcomes may resonate with some employees but be irrelevant or off-putting to others. For example, Ambrose et al. (2008) found that the effect of ethical work climates on employee job attitudes varied as a function of employees’ level of moral development. Similarly, Liao and Rupp (2005) reported that individual differences in employee justice orientation moderated the effect of justice climates on supervisory commitment and satisfaction. Examining work climate as the determinant, Graves et al. (2013) found that environmental leadership moderated the relationship between employee motivation for and frequency of pro-environmental behavior. Person-organization (PO) fit (Kristof, 1996) provides a useful conceptual model for predicting which employees are likely to embrace and flourish under which climates. This is outlined in the next section.

### Person-Environment, Person-Organization, and Green-Person-Organization Fit

Person-environment (PE) fit is defined as “the congruence, match, similarity, or correspondence between the person and the environment” (Edwards and Shipp, 2007, p. 211). Fit can be complementary or supplementary. Complementary fit occurs when a “weakness or need of the environment is offset by the strength of the individual, or vice versa” (Muchinsky and Monahan, 1987, p. 271). This is sometimes referred to as demand-ability fit, given that the specific needs of a situation are fulfilled by a person with the right skill set or ability. Supplementary fit refers to situations where the person and environment possess similar characteristics, such as the case when a culture or work climate is based on values that match those of the people who are living and/or working in that environment (Kristof, 1996). The present study focuses on supplementary fit between organizational climate and employee values as they pertain to pro-environmental outcomes.

PO fit is one type of PE fit that focuses on outcomes arising from the compatibility of employees and the organizations in which they work. Early PO fit research emphasized the extent to which employees’ personalities matched their organizations’ work climate, referred to as personality-climate congruence (Tom, 1971). More recent research has operationalized PO fit in terms of shared values and goals (i.e., value and goal congruence), and also the extent to which organizations provide workplace resources that satisfy employee needs (i.e., need satisfaction; Kristof-Brown et al., 2005). An early meta-analysis by Kristof-Brown et al. (2005) found PO fit to correlate strongly, in a positive direction, with job satisfaction (*r* = 0.41), organizational commitment (*r* = 0.51), and organization satisfaction (*r* = 0.65), and to negatively correlate with quitting intentions (*r* = −0.35).

Green-person-organization (GPO) fit is perhaps best described as a subtype of PO fit that assesses the extent to which an organization’s commitment to environmental protection is congruent with its employees’ environmental values. The concept of GPO fit appears to have originated with Hoffman (1993) who proposed that potential prosperity for “green” organizations may come from understanding more about the influence of a pro-environmental climate at the level of the employee. That is, pro-environmental work climates may have a differential effect on organizational outcomes depending on the extent to which an organization’s environmental values are aligned or misaligned with employees’ environmental values. Previous studies have shown that personal values are associated with pro-environmental behavioral intentions (e.g., de Groot and Steg, 2008, 2010). GPO fit provides the opportunity to not only examine the functional relationship of personal environmental values in the work environment but also determine whether fit effects extend to activity other than environmental protection.

To date, there has been little empirical work investigating the impact of GPO fit on employee and organizational outcomes. Spanjol et al. (2015) found that GPO fit predicted employee job satisfaction, and, in turn, job satisfaction predicted creativity at work. Specifically, value congruence produced greater job satisfaction and more creativity when employees and employers both greatly cared about the environment (high fit) than when both cared little about the environment (low fit). A review of the literature failed to identify any studies that explored the association between GPO fit and employee engagement, defined in this study as commitment to work tasks and intention to remain with one’s current organization. Nor did the review identify any studies investigating the motivational mechanisms through which GPO fit may exert its effects.

A further justification for the current study stems from van Vianen’s (2018, p. 86) review of the PO fit literature. She noted that the expected effect of PO fit on employee job attitudes failed to materialize in some studies and concluded that “some organizational values, such as human relations values, humanity values, and relationships values, are positively related to job attitudes irrespective of employees’ own values.” This finding is particularly relevant to the current study given that it suggests that, at least in some instances, PO fit is less important than the specific values espoused and enacted within an organization’s climate.

A primary aim of the current study is to assess whether pro-environmental values should be added to this list of organizational values that increase employee engagement independently of PO fit. This is not only important from a theoretical perspective, in that it helps define the boundary conditions for PO fit effects, but it may also have important implications for employee recruitment and retention. If a strong corporate commitment to pro-environmental outcomes directly determines employee engagement and retention, unmoderated by employee values, organizations could recruit the most knowledgeable applicants with the strongest skills. However, if fit between organizational climate and employees’ values is a more important determinant of engagement than climate alone, then recruitment should also screen applicants for value congruence.

### Self-Determination Theory and Fit

Although there is compelling evidence that PO fit is positively associated with employee satisfaction and engagement (setting aside the possible boundary conditions identified by van Vianen, 2018), the specific mechanisms through which fit exerts its effects remain unclear. Self-determination theory (SDT; Deci and Ryan, 2000) offers a potential explanatory framework. SDT explicitly links social contexts, such as work climates, to well-being and optimal functioning through the satisfaction of basic psychological (intrinsic) needs. According to SDT, humans are naturally oriented toward satisfying their intrinsic needs for autonomy (i.e., the natural desire to “self-organize experience and behavior and to have activity be concordant with one’s integrated sense of self”; Deci and Ryan, 2000, p. 231), competence (i.e., a sense of proficiency when operating in a particular environment), and relatedness (i.e., the natural inclination to experience a connection with social groups), and these needs are “necessary for healthy development and effective functioning” (Deci and Ryan, 2000, p. 262).

When employee values are congruent with the climate of the organization that employs them (i.e., when PO fit is high), the potential for need satisfaction should be increased. In value-congruent conditions, employees will more likely experience that they are acting with volition and choice, even if their work activities are directed by policies and procedures. Van den Broeck et al. (2016) provide support for this perspective in a recent meta-analysis. Across six studies, they found an average correlation of 0.46 between PO fit and employees’ intrinsic need satisfaction for autonomy, competence, and relatedness. They also found that higher need satisfaction was positively associated with workplace engagement across three subdomains (*r*_autonomy_ = 0.54, *r*_competence_ = 0.33, and *r*_relatedness_ = 0.40) and negatively associated turnover intentions (*r*_autonomy_ = −0.31, *r*_competence_ = −0.05, and *r*_relatedness_ = 0.21), with all effects being statistically significant (*p* < 0.05). In a formal mediation test, Greguras and Diefendorff (2009) integrated PO fit with SDT and found that satisfaction of intrinsic needs partially mediated (explained) the relationship between PO fit assessed as “my personal values match my organization’s values and culture” and organizational commitment. The current study is the first to investigate whether need satisfaction mediates the effect of GPO fit on employee engagement.

### The Current Study

The present study assessed the effect of GPO fit (i.e., the extent to which an organization’s commitment to pro-environmental outcomes is congruent with its employees’ pro-environmental values) on employees’ intrinsic need satisfaction and workplace engagement. Based on our review of the work climate literature, we predicted that employees working in organizations with strong pro-environmental climates would report higher levels of intrinsic need satisfaction and work engagement (Hypothesis 1). In addition, we predicted that these positive work climate effects would increase as a function of GPO fit. That is, we expected that the magnitude of the effects of pro-environmental work climate on employee need satisfaction and engagement would be stronger for employees with pro-environmental value orientations than for employees who are less strongly inclined toward conserving the environment (Hypothesis 2). Finally, based on SDT, we predicted that need satisfaction would mediate the effect of pro-environmental work climate on employee engagement (Hypothesis 3), and the magnitude of this mediation effect would be stronger when GPO fit was high than when GPO fit was low (Hypothesis 4).

## Materials and Methods

### Participants

A community sample of 818 Australian adults participated in this study. All were employed full time when they completed the survey. Women accounted for just over half the sample (52%). Ages ranged from 18 to 69 years: 18–24 (8%), 25–34 (35%), 35–44 (29%), 45–54 (16%), 55–64 (11%), and 65+ years (<1%). The sample included a broad range of education levels: less than year 10 (<1%), year 10 high school (5%), year 12 high school (15%), vocational education training certificate (17%), diploma or advanced diploma (14%), graduate diploma or bachelor degree (34%), and postgraduate university degree (15%). The survey was developed using the Qualtrics™ online survey platform (Provo, UT). Participants were recruited from a Qualtrics research panel and received a small monetary payment for completing the survey. The project was reviewed and approved by the home University’s Human Research Ethics Committee.

### Measures

The survey consisted of measures assessing employee perceptions of workplace pro-environmental climate, ecological worldview, intrinsic need satisfaction, and frequency of work withdrawal behaviors. The survey also included measures of workplace autonomy support, employee motivation to engage in pro-environmental behavior (PEB), and frequency of workplace and non-workplace PEB, which were used for a separate study (Hicklenton et al., 2019). In total, participants responded to 159 items. Cronbach’s alphas reported in this section were based on data from the current study.

#### Demographics

Demographic information was measured and used as control variables in all analyses. Single-item measures assessed participants’ age, gender, and educational attainment.

#### Pro-environmental Work Climate

Employees’ perceptions of their organization’s commitment to positive environmental outcomes were assessed with the Green Work Climate Perception Scale (Norton et al., 2014), with four items, including “Our company is worried about its environmental impact” and “Our company believes it is important to protect the environment.” Participants indicated their agreement with each statement on a scale ranging from 1 (*strongly disagree*) to 5 (*strongly agree*). The items were averaged to compute an overall work climate score in which a high score reflects a perception that the organization is committed to environmental protection. The scale exhibited high internal consistency (Cronbach’s *α* = 0.92).

#### Environmental Values

Participants’ environmental values were assessed using the revised New Ecological Paradigm (NEP) Scale (Dunlap et al., 2000) comprising 15 items and a 7-point scale that ranged from 1 (*strongly disagree*) to 7 (*strongly agree*). High NEP scores reflect ecocentrism, which are defined as recognition that the earth’s carrying capacity is limited and that we are rapidly approaching these limits. Low NEP scores reflect an anthropocentric worldview, which are defined as believing that the earth’s resources should be exploited for human benefit and that our ingenuity as a species will enable us to overcome environmental problems as they arise (Cronbach’s *α* = 0.82).

#### Intrinsic Need Satisfaction

The extent to which participants experience satisfaction of their basic needs was assessed with the Intrinsic Need Satisfaction Scale (Deci et al., 2001). This scale contains 21-items forming three subscales for autonomy, competence, and relatedness based on employees’ experiences on the job during the past year. Representative items include: “I have been able to learn interesting new skills on my job” (autonomy); “On my job I do not get much of a chance to show how capable I am” (competence, reverse scored); and “There are not many people at work that I am close to” (relatedness, reverse scored). Responses were measured using a 7-point scale from 1 (*not at all true*) to 7 (*very true*). Scores on each dimension of the scale were averaged to compute an overall score of intrinsic need satisfaction. A high score reflects positive work experiences, specifically, feeling autonomous, competent, and related to others in the workplace. The decision to use a total need satisfaction score was based on significant intercorrelations (*p* > 0.60) between the autonomy, competence, and relatedness subscales, and previous research that suggests all three subscales predicts employee engagement in the same way. Other researchers (e.g., Van den Broeck et al., 2008) also used an overall score. Cronbach’s *α* for the scale was 0.89, indicating high internal consistency.

#### Employee Engagement

Employee engagement was assessed using the Organizational Withdrawal Scale (Hanisch and Hulin, 1990), which assesses two behavioral aspects of organizational engagement: work withdrawal (the extent to which participants avoid work tasks) and job withdrawal (the frequency with which participants engage in thoughts about behavior related to leaving the organization altogether). The scale contains six items including, “Neglected tasks that wouldn’t affect your evaluation/pay raise” and “Completed work assignments late” for work withdrawal and “Thought about quitting because of work related issues” for job withdrawal. Responses were measured using a 4-point scale from 1 (*once or twice a year*) to 4 (*once a week or more*). Items were reverse scored and then averaged to compute a total workplace engagement score, with higher scores reflecting greater engagement (Cronbach’s *α* = 0.81).

Employee engagement has been defined in many ways (Macey and Schneider, 2008), with some researchers distinguishing between cognitive, affective, and behavioral components (Shuck and Wollard, 2009)^1^.

### Statistical Analyses

All statistical analyses were conducted using SPSS (Version 25). Moderation and mediation tests were conducted using the PROCESS V3.2 macro (Hayes, 2018). Given all hypotheses were directional, 90% confidence intervals and one-tailed significance tests were employed for the moderation and mediation analyses. The survey used a forced response format, so there were no missing data. Examination of boxplots revealed a small number of univariate outliers on most of the variables included in the model but no extreme scores. Three multivariate outliers were identified, and the analyses were re-run with the outliers removed. The re-run analyses generated the same substantive findings with outliers included and excluded. Given that outliers are to be expected in large data sets and that there was no evidence to suggest they were invalid responses, all cases were retained for subsequent analyses reported in this paper.

## Results

### Descriptive Statistics and Preliminary Analyses

Means, standard deviations, and intercorrelations for the main study variables and demographics are presented in Table 1. On average, participants reported that their organizations were moderately committed to environmental sustainability principles and outcomes with the mean on the organizational pro-environmental climate measure falling above the midpoint (3.46 on a 1 to 5 scale). The mean score on the NEP scale also fell above the midpoint (4.87 on a 1 to 7 scale), indicating participants exhibited somewhat stronger levels of ecocentrism than anthropocentrism. On average, participants scored above the midpoint on the intrinsic need satisfaction scale (4.92 on a 1 to 7 scale) and above the midpoint on the (reverse-scored) work withdrawal scale (2.36 on a 1 to 4 scale), indicating they believed their intrinsic needs as individuals were being met at work and they were engaged with their jobs. As is commonly the case in mediation analyses, the correlation between work climate (IV) and engagement (DV) was significant but smaller than the correlation between climate and need satisfaction (the proposed mediator). Gender, age, and education correlated significantly with the theoretical variables in the model and therefore were included as covariates in the moderation and mediation analyses.

**Table 1:** Zero-order correlations and descriptive statistics for study variables (*n* = 818).

Variable	*M*	SD	Correlation (*r*)						
			1	2	3	4	5	6	7
1. Gender	1.54	0.53	—						
2. Age	21.77	11.83	−0.11^**^	—					
3. Education	5.02	1.49	0.00	−0.15^**^	—				
4. Environmental values (ecocentrism)	4.87	0.79	0.19^**^	0.18^**^	0.02	—			
5. Pro-environmental climate	3.46	1.04	−0.06	−0.06	0.17^**^	–0.06	—		
6. Intrinsic need satisfaction	4.92	0.94	0.02	0.06	−0.03	0.01	0.30^**^	—	
7. Employee engagement	2.36	0.70	0.06	0.24^**^	−0.13^**^	0.13^**^	0.08^*^	0.35^**^	—
*Theoretical range for each variable*						1–5	1–7	1–7	1–4

*Point-biserial correlations were computed for all associations involving gender, and Spearman’s *rho* was used for all associations involving education. All other correlations are Pearson’s r*.

* *p < 0.05*.

** *p < 0.01*.

### Moderation Analyses

According to the GPO fit hypothesis, employees with ecocentric values are more likely to have their intrinsic needs satisfied in organizations with strong pro-environmental work climates and also be more engaged with their jobs. To assess these hypotheses, we conducted two moderation analyses using Model 1 in Hayes’ (2018) SPSS PROCESS macro. For both analyses, pro-environmental work climate was the independent variable, and ecocentric values, as assessed by the NEP, were the moderator. Employees’ intrinsic need satisfaction and work engagement were the dependent variables for the first and second analyses, respectively. As recommended by Hayes (2018), both the independent variable and moderator were centered at 0 prior to computing the interaction effect.

In the first moderation analysis, pro-environmental work climate significantly predicted need satisfaction (*B* = 0.26, SE = 0.03, 90% CI = 0.21 to 0.31), but ecocentric values did not (*B* = 0.03, SE = 0.04, 90% CI = −0.03 to 0.10). As predicted, the work climate main effect was qualified by a significant interaction between work climate and ecocentric values (*B* = 0.11, SE = 0.04, 90% CI = 0.06 to 0.17). To probe the significant interaction, we conducted a conditional analysis in PROCESS, assessing the effect of pro-environmental work climate on employee need satisfaction at three levels of ecocentric values: weak (16th percentile), moderate (50th percentile), or strong (84th percentile). This analysis indicated that work climate significantly predicted need satisfaction at all three levels of ecocentrism: (1) weak ecocentrism, *B* = 0.18, SE = 0.04, 90% CI = 0.11 to 0.25; (2) moderate ecocentrism, *B* = 0.26, SE = 0.03, 90% CI = 0.21 to 0.31; and (3) strong ecocentrism, *B* = 0.35, SE = 0.04, 90% CI = 0.29 to 0.42. Consistent with the GPO hypothesis, pro-environmental work climate was a stronger predictor of employee need satisfaction for participants with strong ecocentric values than for those with weak ecocentric values.

**Figure 1:**
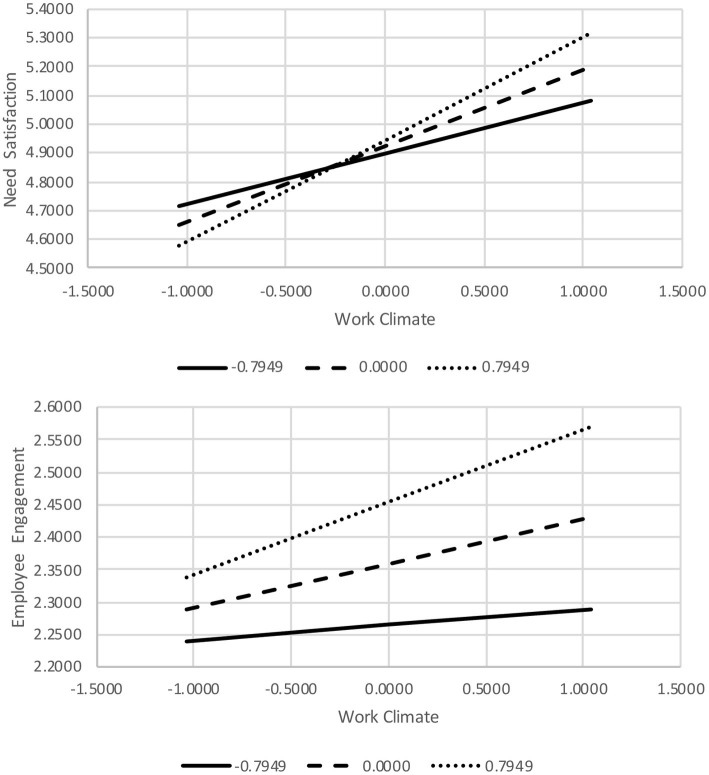
Interaction plots of the predictive effects of work climate on employee need satisfaction and engagement for three levels of environmental values.

In the second moderation analysis, pro-environmental work climate, ecocentric values, and their interaction all significantly predicted employee engagement (*B* = 0.07, SE = 0.02, 90% CI = 0.03 to 0.11 for work climate; *B* = 0.12, SE = 0.03, 90% CI = 0.07 to 0.17 for ecocentrism; and *B* = 0.06, SE = 0.03, 90% CI = 0.01 to 0.10 for the interaction). Once again, a conditional analysis was conducted to probe the interaction. The analysis indicated pro-environmental work climate significantly predicted increased work engagement for employees who scored at moderate (50th percentile, *B* = 0.07, SE = 0.02, 90% CI = 0.03 to 0.11) and 84th percentile (1 SD above the mean, *B* = 0.11, SE = 0.03, 90% CI = 0.06 to 0.16) levels on ecocentric values, but not for employees who scored low (16th percentile, *B* = 0.03, SE = 0.03, 90% CI = −0.03 to 0.08). That is, consistent with the GPO fit model, pro-environmental work climate was a significant predictor of employee engagement for participants with strong ecocentric values, but not for those with weak ecocentric values. Plots for both significant interactions are presented in Figure 1.

### Moderated-Mediation Analysis

The final set of analyses focused on the extent to which intrinsic need satisfaction mediated the predictive effect of pro-environmental work climate on engagement for employees with weak, moderate, and strong ecocentric values. To test these hypotheses, a moderated mediation analysis was conducted using Model 8 within the SPSS PROCESS macro (Hayes, 2018). The analysis revealed that the indirect effect of pro-environmental work climate on engagement, through need satisfaction, was significant for employees with weak (16th percentile, *B* = 0.04, SE = 0.01, 90% CI = 0.02 to 0.068), moderate (50th percentile, *B* = 0.07, SE = 0.01, 90% CI = 0.05 to 0.09), and strong (84th percentile, *B* = 0.09, SE = 0.01, 90% CI = 0.069 to 0.12) ecocentric values. Once again, consistent with the GPO fit model, the indirect effect of pro-environmental climate on engagement, through need satisfaction, was significantly stronger for employees with strong ecocentric values (i.e., when GPO fit was high) than those with weak ecocentric values (i.e., when GPO fit was low), as reflected by the non-overlapping confidence intervals. The direct effect of work climate on worker engagement was non-significant at all three levels of ecocentrism after controlling for the mediator: low (*B* = 0.08, SE = 0.04, 90% CI = 0.01 to 0.16), moderate (*B* = 0.12, SE = 0.03, 90% CI = 0.06 to 0.18), and high (*B* = 0.15, SE = 0.04, 90% CI = 0.08 to 0.21), indicating that need satisfaction fully mediated the effect of climate on engagement.

## Discussion

This study investigated whether GPO fit (the extent to which an organization’s commitment to pro-environmental outcomes is congruent with its employees’ pro-environmental values) would predict employees’ intrinsic need satisfaction and work engagement. Consistent with Hypothesis 1, employees working in organizations with pro-environmental work climates reported higher levels of intrinsic need satisfaction and work engagement. The correlation between work climate and intrinsic need satisfaction was moderate in magnitude, whereas the correlation between work climate and engagement was smaller but still statistically significant. These findings support previous research on positive work climates; organizations’ policies, practices, and procedures that reflect a commitment to corporate responsibility are positively associated with increased employee satisfaction and engagement (Kuenzi and Schminke, 2009). The findings suggest that these effects also apply to organizations with pro-environmental work climates.

To investigate GPO fit, two moderation analyses were conducted. We hypothesized that the positive effects of pro-environmental work climate on intrinsic need satisfaction and workplace engagement would be stronger for employees with strong ecocentric values (i.e., when GPO fit was high) than for those with weak ecocentric values (i.e., when GPO fit was low). This second hypothesis was also supported. Pro-environmental climate was a positive, and statistically reliable, predictor of need satisfaction at all three levels of ecocentrism, but the effect became progressively stronger as a function of employees’ ecocentric values. A similar result was found for employee engagement; pro-environmental work climate became an increasingly stronger predictor of engagement as a function of employees’ ecocentric values. That is, pro-environmental climate failed to predict engagement when employee ecocentrism was low. However, the climate effect increased in magnitude and reached statistical significance at moderate and high levels of ecocentrism, that is, as GPO fit increased.

Importantly, the presence of a pro-environmental climate never became a negative predictor of intrinsic need satisfaction and engagement, even for employees with weak ecocentric values. This suggests that GPO fit may be a more important determinant of need satisfaction and engagement than GPO misfit. Although GPO misfit weakened the positive effect of pro-environmental climate on worker experiences, it did not ever reverse the effect such that having a pro-environmental work climate actually reduced employee engagement, even for employees with non-green value orientations. The effects of GPO fit in the study, on the other hand, were all positive, and the higher the value-congruence between organizations and workers, the greater the benefit.

The study also investigated the process by which GPO fit might influence worker engagement by investigating need satisfaction as a potential mediator. Intrinsic need satisfaction is a central concept from SDT and has been identified as a key determinant of employee motivation and engagement (Deci and Ryan, 2015). Consistent with Hypothesis 3, our results indicate that intrinsic need satisfaction fully mediated the effect of pro-environmental climate on employee engagement. Although this mediation effect held for all participants, regardless of whether they had low, moderate, or high ecocentric values, the indirect effect was significantly stronger when employees’ ecocentric values were high as opposed to low, a finding that is consistent with Hypothesis 4.

### Practical Implications

Our results indicate that high GPO fit may be an important contributor to employee motivation and engagement. Given previous research linking employee engagement to organization success (Gagné and Panaccio, 2015), organizations should consider strategies for increasing GPO fit. This could be done by recruiting new employees based on person-organization-value-congruence and post-hire with training and workshops. Typically, recruiters use person-job fit to determine whether an applicant’s knowledge, skills, and abilities fit with a specific job (Adkins et al., 1994). Instruments have also been designed to assess whether the job applicant and the organization align on various values such as being aggressive, competitive, or supportive (O’Reilly et al., 1991). GPO fit might provide another concrete assessment of value congruence useful in recruitment, particularly for organizations introducing or expanding their pro-environmental policies and procedures.

Socialization and training activities have been shown to increase employees’ perceptions of PO fit (Autry and Wheeler, 2005). As a rule, socialization in a work context is a one-way process in which the purpose of the training is for the organization to transmit information to employees about organizational values and expected role behaviors necessary for employees to be successful in their jobs (Autry and Wheeler, 2005). However, opportunities for two-way socialization should not be overlooked, in which the organization not only conveys environmental information but also recognizes the knowledge and skills of employees and accounts for their concerns about environmental protection, for example, through workshops designed with the purpose of generating interest in environmental protection and supporting employees to integrate environmental tasks with their other work tasks. This might involve allowing employees and work groups to choose how they prioritize different actions aligned to corporate environmental goals (Ryan and Deci, 2000). Where misalignment in environmental values is identified, the purpose of training would be to support employees to internalize corporate environmental values, for example, with information about why the organization has adopted pro-environmental policies and procedures.

### Limitations and Future Research

This study had several limitations that should be considered when interpreting our findings. First, our study relies on self-reported data provided by employees recruited from a non-probability sample. Although we employed a large, diverse national sample, findings cannot presume to be generalizable to the broader Australian population or to other countries. To evaluate the robustness of our findings, we recommend additional studies using a variety of samples, including those from other countries and cultures, and recruited in ways other than through an online panel. We also recommend collecting information using more objective measures of work climate (e.g., independent analysis of organizational policies) and employee engagement (e.g., data from HR on employee performance and turnover).

A second limitation of this study is that it employed a correlational research design. Although mediation analysis implies a causal explanation (Hayes, 2018), in the present study, it should not be used to make strong causal claims. For example, although our mediation analysis provided evidence consistent with the widely held view that work climate causes need satisfaction and engagement, given that all our measures were based on employees’ self-reported perceptions, it is possible that, for example, perceptions of engagement influence perceptions of climate, and not vice versa. It is also possible that the work climate effects observed in the study were due to other uncontrolled organizational variables that covary with pro-environmental climate. For example, it is possible that organizations with strong-pro-environmental climates also have other progressive attributes (such as a commitment to ethics and employee welfare) that are the actual drivers of intrinsic need satisfaction and engagement. Future studies should control for these other factors to rule out possible alternative explanations.

Finally, the current study employed a cross-sectional design, whereby all data were collected at a single time point. It would be beneficial for future GPO work to explore how fit can change over time and which factors drive this change.

### Conclusions

The results of the current study extend previous research on work climate, PO fit, and SDT by demonstrating that (1) organizations with pro-environmental policies, procedures, and processes had more satisfied and engaged employees; (2) the positive effects of pro-environmental work climates were particularly pronounced for employees with pro-environmental values, that is, when GPO fit was high; and (3) employees with pro-environmental values working in organizations with pro-environmental work climates were more engaged because such working environments help them satisfy their intrinsic needs. Overall, our findings highlight the benefits to organizations of implementing pro-environmental policies, procedures, and processes and an added advantage of striving for value congruence between employers and employees.

## Data Availability Statement

The datasets generated for this study are available on request to the corresponding author.

## Ethics Statement

The study was reviewed and approved by the University of New England Human Research Ethics Committee. The participants provided their written informed consent to participate in this study.

## Author Contributions

CH and DH conceived and designed the analysis. CH collected the data. CH and DH performed the analysis. CH, DH, and NL wrote the paper.

### Conflict of Interest

The authors declare that the research was conducted in the absence of any commercial or financial relationships that could be construed as a potential conflict of interest.

## Footnotes

^1^In the current study, we chose to focus on behavioral engagement, rather than the cognitive and affective components, given that we (1) were primarily interested in the practical behavioral outcomes of GPO fit and (2) wanted to minimize the conceptual overlap with the perceived work climate and need satisfaction variables in our model.
